# Stigmatizing weight experiences in health care: Associations with BMI and eating behaviours

**DOI:** 10.1002/osp4.379

**Published:** 2019-11-12

**Authors:** Jocelyn E. Remmert, Alexandra D. Convertino, Savannah R. Roberts, Kathryn M. Godfrey, Meghan L. Butryn

**Affiliations:** ^1^ Center for Weight, Eating, and Lifestyle Science (WELL Center), College of Arts and Sciences Drexel University Philadelphia Pennsylvania; ^2^ San Diego Joint Doctoral Program in Clinical Psychology San Diego State University/University of California San Diego California; ^3^ Joint Doctoral Program in Clinical and Developmental Psychology University of Pittsburgh Pittsburgh Pennsylvania

**Keywords:** behavioural weight loss, delivery of health care, eating behaviour, weight stigma

## Abstract

**Introduction:**

Individuals with overweight or obesity often experience stigmatizing weight‐related interactions in health care, though how these experiences are associated with body mass index (BMI) and eating behaviour is unknown. This study had three aims: (a) characterize types and frequency of stigmatizing health care experiences, (b) assess relationships among BMI, eating behaviour, and stigmatizing experiences, and (c) examine whether internalized weight stigma mediates the relationship between stigmatizing experiences, weight, and eating behaviour.

**Methods:**

Adults (N = 85) enrolled in behavioural weight loss completed measures of stigmatizing health care experiences, weight bias internalization, eating behaviours, and BMI. Cross‐sectional correlational and mediational analyses were conducted.

**Results:**

The majority (70.6%) of participants reported at least one stigmatizing health care experience in the past year. Greater amounts of stigmatizing experiences were associated with higher BMI (*r* = 0.32, *P* < .01) and greater uncontrolled (*r* = 0.22, *P* = .04) and emotional eating (*r* = 0.28, *P* < .01). Internalized weight stigma significantly mediated the relationship between stigmatizing experiences and maladaptive eating.

**Conclusion:**

Experiences of health care weight stigma were associated with eating behaviour and BMI. Participants with a higher BMI or greater maladaptive eating behaviours may be more susceptible to stigmatizing experiences. Reducing internalized weight stigma and health care provider stigma may improve patient health outcomes.

## 1 INTRODUCTION

Given the high prevalence and negative health consequences of obesity, professional guidelines recommend that medical providers (eg, physicians and nurses) discuss weight, give advice to lose weight, and offer behavioural interventions to support behaviour change.^1^ Unfortunately, patients with overweight or obesity often have stigmatizing experiences when receiving medical care, which impacts the care they receive and their engagement in the medical system.^2^ Health care providers often harbour both explicit^3^ and implicit^4^ biases towards their patients with overweight or obesity, resulting in negative health care experiences for patients.^5^, ^6^ Broadly, weight stigma is when individuals with overweight or obesity are devalued in society, leading to prejudice, negative stereotyping, and discrimination.^7^ When interacting with health care providers, this can take the form of explicit, derogatory comments,^8^ insensitive language,^9^ or more subtle interactions that a patient may perceive as stigmatizing, such as a provider attributing medical problems to a patient's weight when the patient does not perceive there to be a link between weight and the medical problem.^10^ Furthermore, patients report receiving overly simplistic recommendations for weight loss from providers, which they perceive to be insensitive and humiliating (eg, “eat less, move more” as the only recommendation).^10^, ^11^ Negative weight‐related experiences in health care (ie, all providers, staff, equipment, and facilities) are common and have measurable health consequences for individuals with overweight or obesity, including reduced perception of provider empathy,^12^ reduced success in weight loss programmes,^13^ and missing or delaying medical appointments.^2^, ^14^

Experiencing weight‐based stigma generally is associated with a range of negative emotional states, including depressive symptoms, anxiety symptoms, and lower self‐esteem.^15^, ^16^ Stigmatizing experiences are also significantly associated with negative behavioural outcomes, including eating disturbances, such as binge eating^16^, ^17^ and emotional eating.^18^, ^19^ In addition, longitudinal studies have found that weight‐related discrimination is associated with greater risk of weight gain and obesity.^20^, ^21^, ^22^ Hypothesized mechanisms in this process include increased caloric intake^23^, ^24^ and hypercortisolism^25^ in response to experienced stress and stigma. While general weight‐based stigma experiences have a robust association with maladaptive eating behaviours, little is known about the relationship between stigmatizing experiences in health care specifically and weight and eating behaviour.

A suggested explanatory factor in the relationship between stigmatizing experiences and maladaptive eating patterns and/or weight gain is internalized weight stigma. Internalized weight stigma is when individuals internalize negative weight‐based stereotypes and subsequently devalue themselves because of their weight.^26^ Internalized weight stigma, independent of experienced discrimination, is associated with negative outcomes. Specifically, greater internalized weight stigma is associated with greater difficulty adhering to weight control behaviours, lower likelihood of maintaining weight loss, and greater amounts of uncontrolled eating.^27^, ^28^, ^29^, ^30^, ^31^ In conjunction with stigmatizing situations, internalized weight stigma appears to mediate the relationships between stigmatizing weight‐related experiences and maladaptive cognitive and psychological outcomes. In a large study of individuals with overweight or obesity, having a greater number of stigmatizing experiences was associated with higher internalized weight stigma, which in turn predicted a higher number of maladaptive coping mechanisms (eg, negative self‐talk and isolation), which then predicted higher levels of depression, anxiety, and stress symptoms.^32^ Taken together, these findings demonstrate the importance of investigating the role of internalized weight stigma in the relationships between stigmatizing situations and maladaptive behaviours, particularly in a treatment‐seeking population, whose unique experiences have thus far been understudied.

Individuals participating in a behavioural weight loss treatment are a particularly relevant group in which to study the relationship between stigmatizing situations and weight and eating behaviours. In behavioural weight loss samples, experiencing higher amounts of stigmatizing situations broadly has been found to predict greater amounts of binge eating, greater programme attrition, poorer programme adherence, and poorer weight loss outcomes.^33^, ^34^ Problematic eating behaviours, such as emotional eating and binge eating, are commonly cited barriers to both continued engagement in weight loss programmes and weight loss success.^35^, ^36^, ^37^, ^38^ Little is known about the relationship between negative experiences in health care specifically and these behaviours, which could be important for clinicians providing behavioural weight loss treatment to understand. Participants may enter behavioural weight loss with the belief that most health care providers, including their behavioural weight loss clinicians, have weight stigma. If participants have this perception upon entering treatment, it may impact the quality of the therapeutic relationship between participant and clinician, potentially impairing treatment adherence and weight loss outcomes.^39^ Therefore, understanding mechanisms of action that are linked to maladaptive eating behaviours, and thus weight gain, could help inform future weight loss interventions, improve treatment retention, and enhance treatment outcomes for participants experiencing weight stigma.

Overall, there is widespread prevalence of weight stigma in health care. However, the relationship between stigmatizing experiences in health care and weight and maladaptive eating behaviours is less clear, despite the potential importance of these variables for participants in behavioural weight loss programmes. After reviewing relevant literature, it appears that no study to date has examined stigmatizing experiences in health care in a behavioural weight loss sample. Therefore, the first aim of this study is to describe the quantity and types of stigmatizing experiences in health care reported by this sample. The second aim is to examine associations between the amount of stigmatizing health care experiences reported by participants and weight and eating behaviour. Additionally, given the evidence that internalized weight stigma may explain the relationship between general stigmatizing situations and maladaptive behaviours, the third aim is to test the hypothesis that internalized weight stigma mediates the relationship between stigmatizing health care experiences and weight and eating behaviour.

## 2 METHODS

### 2.1 Participants

This is a secondary data analysis conducted with participants originally recruited from the community for a clinical trial of group‐based behavioural weight loss (R21DK112741). Eligible participants were adults age 18 to 70 years with a body mass index (BMI) of 25 to 45 kg/m^2^, had a smartphone and home wireless access, and were able to engage in physical activity. Table 1 contains demographic and variable information. Participants were excluded if they had a medical or psychiatric condition that may have posed a risk to their participation in the behavioural weight loss intervention, had recently began or changed the dose of a medication that could cause significant change in weight, had received bariatric surgery, or had a weight loss of greater than or equal to 5% in the previous 3 months. Informed consent was obtained from all participants prior to any study procedures. This analysis focused on the baseline of the parent clinical trial, utilizing only measurements taken prior to any randomization or intervention. All procedures were approved by the Institutional Review Board at Drexel University.

**Table 1:** Participant demographics

Characteristic	
Gender	
Female, n (%)	70 (82.4%)
Male, n (%)	15 (17.6%)
Race	
White, n (%)	43 (50.6%)
Asian, n (%)	3 (3.5%)
Black or African American, n (%)	31 (36.5%)
Other or more than one race, n (%)	8 (9.4%)
Age, M (SD)	50.3 (12.8)
BMI, M (SD)	34.9 (4.9)
WBIS, M (SD)	3.7 (1.1)
SSHC, M (SD)	3.4 (5.7)
TFEQ uncontrolled eating, M (SD)	38.2 (18.9)
TFEQ emotional eating, M (SD)	55.0 (22.9)
TFEQ cognitive restraint, M (SD)	46.3 (15.8)

Abbreviations: BMI, body mass index; SSHC, Stigmatizing Healthcare Situations in Healthcare stigma score; TFEQ, Three‐Factor Eating Questionnaire; WBIS, Weight Bias Internalization Scale.

### 2.2 Measures

Participant height and weight were measured twice using a Tanita model WB‐3000 scale and then averaged by study staff. Participants reported demographics in the Weight and Lifestyle Inventory (WALI).^40^

The Stigmatized Situations in Healthcare (SSHC)^12^ instrument is a 20‐item scale with good reliability (Cronbach α = .89) for individuals with overweight or obesity that was used to assess how often an individual experienced different weight‐stigmatizing situations during a health care appointment in the past 12 months (eg, “A doctor blaming unrelated physical problems on your weight,” see Table 2 for all items listed). The SSHC is used to calculate an “overall stigma” score, found by summing all items. Each item used a 4‐point Likert scale score calculated on the basis of the frequency of the stigmatizing experience: never (0 points), one time (1 point), two times (2 points), and multiple times in the past 12 months (3 points). Of note, the scoring format was slightly modified for the present study because the SSHC included anchors that were intended for after a medical appointment, rather than at a research assessment not immediately following such an appointment.

**Table 2:** Stigmatizing experiences in health care reported by sample in the past year

How often has this happened to you in the past 12 months?	At least once n (%)
A doctor saying weight is a health problem when you are in good health.	33 (38.8)
A doctor blaming unrelated physical problems on your weight.^a^	32 (38.1)
A doctor recommending a diet even if you did not intend to discuss weight.	29 (34.1)
A doctor telling you to lose weight but not providing weight loss treatment options or advice on how to get help for weight loss.	29 (34.1)
Having health care professionals make suggest diets to you without you asking for advice.	14 (16.5)
Having doctors or other health professionals assume you overeat or binge because you are overweight.	11 (12.9)
Not being able to find medical equipment, such as blood pressure cuffs or gowns that fit you.	7 (8.2)
Having doctors or other health professionals assume you have emotional problems because you are overweight. a	6 (7.1)
Being treated as less competent by your health care providers because of your weight.	6 (7.1)
Being treated as lazy by your health care providers because of your weight. a	6 (7.1)
A doctor makes cruel remarks, ridicules you or calls you names.	4 (4.7)
Being stared at by medical staff when you go to the doctor's office. a	4 (4.7)
Overhearing medical staff make rude comments to you. a	3 (3.6)
When you are weighed on a scale, the medical staff member makes negative comments about your weight.	2 (2.4)
When you are weighed on a scale, the scale is not large enough for your size.	1 (1.2)
Having nurses make negative remarks, ridicule you, or call you names.	1 (1.2)
Having office staff, for example a front desk receptionist make negative remarks to you about your weight. a	1 (1.2)
Not being able to fit in chairs at the waiting room. a	1 (1.2)
A doctor refusing to do an exam on you because of your weight. a	1 (1.2)
Having medical staff make negative comments about weight to others. a	1 (1.2)

a Missing n = 1 participant data. Percentages calculated out of n = 84; Imputed data not used in this table to reflect actual reported rates.

Internalized weight bias was assessed using the Weight Bias Internalization Scale (WBIS), an 11‐item scale with demonstrated reliability and validity in adults with overweight or obesity (Cronbach α = .85).^41^ The WBIS asked participants their agreement with statements reflecting weight bias (eg, “I am less attractive than most other people because of my weight”) using a 7‐point Likert scale ranging from strongly disagree (1) to strongly agree (7). Items are summed then averaged, with higher scores indicating higher weight bias internalization.

The revised 18‐item Three‐Factor Eating Questionnaire (TFEQ‐R18)^42^ was used to assess uncontrolled eating, cognitive restraint, and emotional eating (cognitive restraint Cronbach α = .61, uncontrolled eating Cronbach α = .85, and emotional eating Cronbach α = .77). The TFEQ‐R18 asks participants to provide their agreement with a provided eating behaviour or cognition (eg, “When I smell a sizzling steak or juicy piece of meat, I find it very difficult to keep from eating, even if I have just finished a meal”) on a 4‐point Likert scale ranging from definitely false (1) to definitely true (4). Raw scale scores are transformed into a 0 to 100 scale; higher scores are indicative of greater cognitive restraint, uncontrolled eating, or emotional eating.

### 2.3 Statistical analyses

SPSS version 25 (*IBM* Corp. *Armonk*, *NY)* was utilized for all analyses. Descriptive data are presented for the first aim. Pearson correlation coefficients were utilized to examine associations at baseline (aim 2). In addition, a linear regression was utilized to examine associations between stigmatizing situations in health care and eating behaviours (uncontrolled and emotional eating) at baseline while controlling for BMI (aim 2). The PROCESS Macro^43^ was utilized for mediation analyses (aim 3). In the mediation analyses, race, gender, and BMI were included in covariates. Bootstrapped 95% confidence intervals (CIs) of 5000 samples were calculated by the PROCESS Macro and utilized when examining indirect effects. The parent study enrolled 87 participants. However, the current study retained 85 because of the SSHC not being completed by two participants. Nine participants were missing one item each on the SSHC. One participant had two items missing on the WBIS. Missing data were replaced by an average of the other items in the scale.

## 3 RESULTS

Participants (n = 85) were on average 50.3 years old (SD = 12.8 years) with an average BMI of 34.9 kg/m^2^ (SD = 4.9). The majority of the sample was female (82.4%) and white (50.6%) or black/African American (36.5%). See Table 1 for demographic and variable information.

### 3.1 Aim 1: Quantity and types of stigmatizing experiences

Over two‐thirds (70.6%) of participants reported at least one instance of a stigmatizing health care experience in the past 12 months. The average SSHC score was 3.4 (SD = 5.7). The top four most frequently endorsed items (in descending order) were the following: a doctor saying weight is a health problem when you are in good health (38.8%); a doctor blaming unrelated physical problems on your weight (38.1%); a doctor recommending a diet even if you did not intend to discuss weight (34.1%); and a doctor telling you to lose weight but not providing weight loss treatment options or advice on how to get help for weight loss (34.1%). See Table 2 for additional information.

### 3.2 Aim 2: Associations between stigma, weight, and eating behaviours

Having a higher SSHC score was associated with higher BMI (*r* = 0.32, *P* = .003) and greater levels of uncontrolled (*r* = 0.22, *P* = .04) and emotional eating (*r* = 0.28, *P* = .01) but was not associated with cognitive restraint (*r* = −0.14, *P* = .2) (Table 3). The relationship between the SHCC score and uncontrolled and emotional eating remained significant when BMI was controlled for in each regression model (uncontrolled eating: *F*_2,82_ = 7.72, *P* = .001, *R* ^2^ = 0.14; emotional eating: *F*_2,82_ = 7.60, *P* = .001, *R* ^2^ = 0.16).

**Table 3:** Correlation matrix of key variables

Measure	1	2	3	4	5	6	7	8	9
1. Age	‐	0.05	0.02	−0.09	−0.18	−0.07	−0.23	0.12	−0.13
2. BMI		‐	0.12	−0.05	0.14	0.32**	−0.05	−0.14	0.15
3. Race			‐	−0.33*	−0.3**	−0.10	−0.32**	−0.03	−0.33**
4. Gender				‐	0.02	0.16	0.19	0.05	0.03
5. WBIS					‐	0.31**	0.5**	−0.17	0.53**
6. SSHC						‐	0.22*	−0.14	0.28*
7. TFEQ uncontrolled eating							‐	−0.18	0.62
8. TFEQ cognitive restraint								‐	−0.13
9. TFEQ emotional eating									‐

*Note*. Race was recoded dichotomously for analysis, 0 was white and 1 was non‐white; Gender was coded such that 0 represented female and 1 represented male.

Abbreviations: BMI, body mass index; SSHC, Stigmatizing Healthcare Situations in Healthcare score; TFEQ, Three‐Factor Eating Questionnaire; WBIS, Weight Bias Internalization Scale.

* *P* < .05.

** *P* < .01.

### 3.3 Aim 3: Mediation

**Figure 1:**
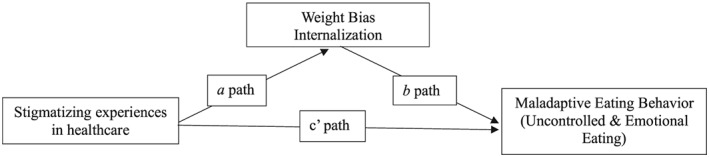
Mediation model, controlling for race, gender, age, and body mass index (BMI)

The final aim tested the hypothesis that internalized weight stigma mediates the relationship between the SSHC score and weight and eating behaviour. See Figure 1 for overall mediation model and labelled paths. Internalized weight stigma did significantly mediate the relationship between the SSHC score and uncontrolled eating when controlling for race, gender, BMI, and age (indirect effect *b* = 0.36; Boot SE: 0.19; 95% Bootstrapped CI, 0.01 to 0.75) (see Table 4). This was full mediation as the *c*′ path was not significant in the mediation model (*c*′ path: *b* = 0.29, SE = 0.34, *P* = .40).

**Table 4:** Mediation analysis testing if weight bias internalization mediated the relationship between stigmatizing situations in health care and uncontrolled eating, controlling for BMI, gender, age, and race, paths as labelled in Figure 1

	*a* path			*b* path			*c′* path		
	Coeff.	SE	*P*	Coeff.	SE	*P*	Coeff.	SE	*P*
SSHC	0.05	0.02	.02	‐	‐	‐	0.29	0.34	.40
Race	−0.73	0.24	.003	−5.14	3.94	.20	‐	‐	‐
Gender	−0.41	0.31	.19	4.87	4.97	.33	‐	‐	‐
Age	−0.02	0.01	.08	−0.19	0.14	.17	‐	‐	‐
BMI	0.02	0.02	.40	−0.42	0.39	.28	‐	‐	‐
WBIS	‐	‐	‐	6.91	1.77	.0002	‐	‐	‐

*Note*. Indirect path *ab*: Coeff. = 0.36; Boot SE: 0.19; 95% Bootstrapped CI, 0.01 to 0.75.

Abbreviations: BMI, body mass index; CI, confidence interval; Coeff., coefficient; SE, standard error; SSHC, Stigmatizing Situations in Healthcare; WBIS, Weight Bias Internalization Scale.

Internalized weight stigma also significantly mediated the relationship between SSHC score and emotional eating (indirect effect *b* = 0.43; Boot SE: 0.20; 95% Bootstrapped CI, 0.02 to 0.81) (Table 5). Similarly, there was total mediation as *c*′ path was not significant in the mediation model (*b* = 0.45, SE = 0.41, *P* = .28). Internalized weight stigma did not mediate the relationship between the SSHC score and BMI when controlling for race, gender, and age (indirect effect *b* = 0.03; Boot SE: 0.03; 95% Bootstrapped CI, −0.04 to 0.08).

**Table 5:** Mediation analysis testing if weight bias internalization mediated the relationship between stigmatizing situations in health care and emotional eating, controlling for BMI, gender, age, and race, paths as labelled in Figure 1

	*a* path			*b* path			*c′* path		
	Coeff.	SE	*P*	Coeff.	SE	*P*	Coeff.	SE	*P*
SSHC	0.05	0.02	.018	‐	‐	‐	0.45	0.41	.28
Race	−0.73	0.24	.003	−10.11	2.13	.04	‐	‐	‐
Gender	−0.41	0.31	.19	−4.52	5.98	.45	‐	‐	‐
Age	−0.02	0.01	.08	−0.10	0.17	.57	‐	‐	‐
BMI	0.02	0.02	.40	0.35	0.47	.45	‐	‐	‐
WBIS	‐	‐	‐	8.44	2.13	.0002	‐	‐	‐

*Note*. Indirect path *ab*: Coeff. = 0.43; Boot SE: 0.20; 95% Bootstrapped CI, 0.02 to 0.81.

Abbreviations: BMI, body mass index; CI, confidence interval; Coeff, coefficient; SE, standard error; SSHC, Stigmatizing Situations in Healthcare; WBIS, Weight Bias Internalization Scale.

## 4 DISCUSSION

This study examined the relationship between stigmatizing experiences in health care and weight and eating behaviour. The majority of participants reported at least one stigmatizing experience in health care in the past year, and the average stigma score was 3.4 (SD = 5.7). As compared with a broader sample of individuals with overweight or obesity presenting to a doctor's office, for a typical appointment and not for weight loss specifically, this sample did report a lower stigma score in the past year.^12^ There were several key differences between these samples that may explain the differences, including the present sample having an average BMI of 34.9 kg/m^2^ compared with an average BMI of 39.4 kg/m^2^ in the Ferrante et al^12^ sample. Overall, the current sample reported very few overt stigmatizing experiences (eg, getting ridiculed) and many subtler stigmatizing experiences (eg, a provider bringing up weight when an individual did not think it was relevant to their health). These actions may be enacted by well‐meaning providers who want to provide advice, but to patients who have overweight or obesity, providers offering simple solutions to a complex issue can be perceived as insensitive.^11^ While it is promising that patients reported low amounts of overt weight discrimination in health care overall, this study suggests that patients are experiencing more subtle forms of weight bias in health care that could be impacting their clinical care.

Previous work has demonstrated that there is significant implicit weight bias even among health care providers who specialize in obesity.^44^ Pervasive implicit weight bias may contribute to more subtle forms of weight stigma, such as microaggressions, which have been shown in other domains to have a serious impact on health and well‐being.^45^, ^46^, ^47^ Providing basic and ongoing training in obesity medicine with a focus on reducing stigma is likely to be helpful to improve patient health care overall.^48^

Given the measure utilized in this study, it is also possible that these instances are a subjective interpretation of a stigmatizing situation from the patient point of view. For example, the second most frequently endorsed item, “a doctor blaming unrelated physical problems on your weight,” may stem from a patient not understanding—either because of lack of knowledge on the part of the patient or lack of provider explanation—that a variety of medical issues are related to weight, such as sexual function, fertility, chronic pain, and cancer.^49^, ^50^, ^51^, ^52^, ^53^, ^54^ Therefore, a doctor may reasonably bring up the topic of weight in an appointment to address some modifiable lifestyle factors affecting these concerns, but the patient may interpret this suggestion as unprompted and stigmatizing. The impact of these types of experiences on the patient may still be powerful and negative; however, the experiences and the stigma felt from them may originate from a point of misunderstanding rather than direct provider enacted stigma. In order to prevent these stigmatizing situations from occurring, health care providers ought to take special precaution in detailing the medical relationship between excess weight and the health condition they are addressing with their patient. Future studies should seek to gather more detailed data to better understand different types of stigma and perception of stigma and how these may have different relationships with outcomes.

Greater reported number of stigmatizing situations in health care was associated with higher BMI and more severe uncontrolled and emotional eating. Previous research has examined the relationship between stigmatizing experiences generally and a range of cognitive, behavioural, and biological outcomes including emotional eating,^18^, ^19^ binge eating,^16^, ^17^ negative mood,^15^, ^16^ and increased weight gain.^20^, ^21^, ^22^ Additionally, while previous literature clearly demonstrated that weight bias in health care is highly prevalent, little is understood about the relationship between this weight bias in health care and patient health outcomes.^5^, ^55^ In the present study, it is apparent that reporting experiencing weight bias in health care settings is associated with negative health behaviours, such as uncontrolled and emotional eating, particularly since this relationship holds even when BMI is controlled for. These eating behaviours have been shown to affect behavioural weight loss treatment in previous studies. For example, higher rates of these maladaptive eating behaviours are associated with higher rates of dropout from treatment and less weight loss.^35^, ^36^, ^37^, ^38^ Behavioural weight loss providers should be aware that previous stigmatizing situations in health care may be a marker of risk for suboptimal treatment outcomes,^13^ although additional longitudinal research is required to fully understand these relationships.

Internalized weight stigma fully mediated the relationship between stigmatizing experiences in health care and emotional and uncontrolled eating, such that having a higher score of stigmatizing situations was associated with higher internalized weight stigma, which in turn was associated with higher levels of maladaptive eating behaviours. The pattern of findings across mediation models suggests that internalized weight stigma fully mediated the relationship between stigmatizing experiences in health care and these eating behaviours. One interpretation of these results is when health care providers perpetuate weight stigma, individuals with higher internalized weight stigma may experience greater emotional and uncontrolled eating as a result, but without experimental manipulation, any causal relationship between these variables is only speculative.

Because of the cross‐sectional nature of this study, it is important to consider other explanations for these relationships. Specifically, it is possible that individuals with a higher BMI or more problematic eating behaviours interpret health care experiences differently and may be more vulnerable to or more aware of weight stigma. This may be particularly true in the present study because of the most frequently endorsed stigmatizing experiences being more subjective in nature. Behavioural weight loss and health care providers should be aware that patients with higher weights and/or higher amounts of problematic eating behaviours may have a greater weight stigma history or a lower threshold for perceiving situations as stigmatizing. Providers may wish to be particularly careful with these patients to not further stigmatize them. In clinical practice, providing nonstigmatizing care starts from when the patient or participant enters the office, including adequate office equipment (eg, chairs without arms) and a private weighing area. As with any other medical procedure or recommendation, patients can opt out of being weighed if they desire. In the appointment, health care providers should prioritize the topics the patient is interested in addressing and treat weight as one piece of information in the context of overall health (ie, not over emphasizing the importance of weight vs another measure, such as blood pressure). Should a provider believe it necessary to address the patient's weight, they should ask if their patient is interested in discussing their weight, prior to giving recommendations. Additional recommendations can be found in Phelan et al.^5^

These findings underscore the importance of both reducing stigmatizing situations in health care and reducing patients perceiving ambiguous situations as stigmatizing. In regards to reducing of stigmatizing situations in health care, previous interventions have sought to address health provider weight stigma to improve the quality of weight‐related communication.^56^ Interventions that deliver education on the genetic and environmental effects on obesity, implementation of a brief calorie restricted diet (the health care provider was placed on the diet), and longitudinal contact with a patient with obesity have had some small‐scale success in addressing health care provider weight stigma.^57^, ^58^, ^59^ However, there is no robust intervention to address health care provider weight stigma to improve the quality of weight‐related communication.^56^ Future studies should continue to determine how to reduce weight stigma in health care settings and evaluate how that may improve health care.

The present study also suggests that internalized weight stigma explains the relationship between stigmatizing situations and eating behaviours. It may be beneficial to directly address internalized weight stigma and thus potentially address some of the beliefs that may lead a patient to interpret an ambiguous situation as stigmatizing. A pilot study demonstrated that eight sessions of cognitive behavioural therapy could produce a significant reduction in internalized weight stigma as compared with a quasi‐control group.^60^ Future behavioural weight loss treatments may consider adding treatment components that specifically help patients recognize and target the negative effects of stigma on their health care utilization, health behaviours, and outcomes. It may also be beneficial for providers to consistently provide psychoeducation prior to recommending weight loss to avoid misunderstandings. Future interventions could seek to concurrently reduce internalized weight stigma in patients and stigma in health care providers to address both potential negative pathways of these factors with weight and eating behaviours.

Internalized weight stigma did not explain the relationship between stigmatizing experiences in health care and BMI. Prior research has not found a clear, consistent relationship between BMI and internalized weight stigma in samples with overweight and obesity.^26^ This is likely due to the fact that there are a multitude of factors that affect weight, including, but not limited to, genetics, physiology, and environment.^61^ Additional research is needed to better understand the relationship between internalized weight stigma and BMI, particularly in the context of stigmatizing experiences in health care.

This study has several limitations. The analyses are cross‐sectional; therefore, causation among the variables cannot be established. Another limitation of this study is the lack of general stigma measurement. This study sought to examine health care stigma specifically, a novel contribution. However, health care stigma is only one type of weight stigma that people can experience. Past research has demonstrated a relationship between general stigma and maladaptive eating behaviours, and this study may be capturing general stigma experienced in the measurement of health care experience.^33^, ^34^ A limitation in the mediation model is that the order of the variable implies that internalized weight stigma may come after the stigmatizing experience in health care. There is evidence that weight bias internalization can occur in the absence of stigma, and therefore, the use of the mediation model implying that weight bias internalization is only due to enacted stigma is a limitation of the present study.^26^

Additionally, this sample was primarily comprised mostly white, middle‐aged females, which may limit generalizability. Previous work has demonstrated that internalized weight stigma may have a larger effect on difficulty with weight loss maintenance in males.^31^ Therefore, further examination of internalized weight stigma and its relationship with stigmatizing situations in health care and eating behaviour by gender is warranted, particularly in longitudinal analyses. Additionally, the present sample demonstrated differences in several variables by race, such that white participants had higher amounts of internalized weight stigma and uncontrolled and emotional eating as compared with non‐white participants. Race was controlled for in mediation analyses, but future studies with a larger sample could explore the effect of race on the relationships examined in the current study. An additional potential limitation is that individuals with overweight or obesity or eating difficulties may interact with health care providers more frequently, providing more potential opportunities for stigmatizing experiences to occur and potentially artificially altering the results. Another limitation is the reliance on self‐report measures. Future studies could potentially further test the relationships between experiencing stigmatizing situations in health care and negative eating behaviour outcomes with a lab‐based buffet study.

This study examined the relationship between stigmatizing experiences in health care and eating behaviour and weight in a behavioural weight loss sample. The majority of participants reported at least one stigmatizing experience in health care in the past year. Reporting a greater number of stigmatizing situations in health care was associated with higher BMI and greater levels of uncontrolled and emotional eating. Participants' internalized weight stigma significantly mediated the relationship between stigmatizing health care experiences and uncontrolled eating and emotional eating, such that having a higher score of stigmatizing situations in health care was associated with higher internalized weight stigma, which was associated with higher levels of maladaptive eating behaviours. These models demonstrated full mediation, as the relationships between stigmatizing experiences in health care and uncontrolled and emotional eating were no longer significant in the model with internalized weight bias.

Additionally, this study links stigmatizing experiences in health care to maladaptive eating behaviours that negatively affect treatment outcomes. Providers should be aware that individuals with a higher weight or higher amounts of maladaptive eating may have a more extensive history of weight stigma in health care settings and should be careful of how this may affect treatment outcomes. Future studies should seek to investigate these relationships in longitudinal analyses to further understand the potential effects of stigmatizing weight‐related experiences in health care.

## CONFLICT OF INTEREST STATEMENT

No conflicts of interest to report.
